# Supplementary material for: Effectiveness and Acceptance of Multimodal Antibiotic Stewardship Program: Considering Progressive Implementation and Complementary Strategies
Source: Antibiotics (Basel). 2020 Nov 27;9(12):848. doi: 10.3390/antibiotics9120848 (PMC7760905; doi:10.3390/antibiotics9120848)
Supplement: Supplementary file 1 [file antibiotics-09-00848-s001.pdf]

antibiotics

Supplementary Materials

Effectiveness and Acceptance of Multimodal Antibiotic Stewardship Program: Considering Progressive Implementation and Complementary Strategies

Flavien Bouchet [1,2,*], Vincent Le Moing [1], Delphine Dirand [2], François Cros [3], Alexi Lienard [4], Jacques Reynes [1], Laurent Giraudon [2] and David Morquin [1]

[1] Service de Maladies Infectieuses et Tropicales, Centre Hospitalier Universitaire de Montpellier, Université de Montpellier, 34000 Montpellier, France; v-le_moing@chu-montpellier.fr (V.L.M.); j-reynes@chu-montpellier.fr (J.R.); d-morquin@chu-montpellier.fr (D.M.)
[2] Pôle Appui aux Fonctions Cliniques, Département de la Pharmacie, Hôpitaux du bassin de Thau, Sète, 34200 Sète, France; ddirand@ch-bassindethau.fr (D.D.); lgiraudon@ch-bassindethau.fr (L.G.)
[3] Département Informatique, Hôpitaux du Bassin de Thau, Sète, 34200 Sète, France; fcros@ch-bassindethau.fr
[4] Département de Biologie Médicale, Hôpitaux du Bassin de Thau, Sète, 34200 Sète, France; alienard@ch-bassindethau.fr
* Correspondence: fbouchet@ch-bassindethau.fr

Table S1. Satisfaction survey regarding the antibiotic stewardship program in a tertiary hospital

Question 1: You are satisfied with the availability of this type of intervention within the hospital center

Question 2: This kind of intervention relieves you in your daily practice

Question 3: This kind of intervention is profitable for the patient

Question 4: Notification in electronic patient record is very satisfactory

Question 5a: Infectious disease specialist is easy to reach for this kind of intervention

Question 5b: I am satisfied with the systematic phone call of the infectious disease specialist just after the notification in electronic patient record

Question 6: The therapeutic or diagnosis proposal noted in the electronic patient record is sufficiently understandable

Question 7: This kind of intervention interfering with your medical practice

Question 8: It would be interesting to continue this kind of intervention in your hospital

Regarding on-request IDSCS intervention: 49 answers

	Strongly disagree	Somewhat disagree	Somewhat agree	Strongly agree
Question 1			1	48
Question 2		1	12	36
Question 3			5	44
Question 4			5	44
Question 5a		1	9	39
Question 6		1	2	46
Question 7	33	13	3	
Question 8			3	46

Regarding Microbiological laboratory meetings intervention: 49 answers

	Strongly disagree	Somewhat disagree	Somewhat agree	Strongly agree
Question 1	1	[redacted]	6	42
Question 2	[redacted]	2	9	38
Question 3	[redacted]	[redacted]	6	43
Question 4	[redacted]	1	7	41
Question 5b	[redacted]	2	5	42
Question 6	[redacted]	2	6	41
Question 7	30	16	3	[redacted]
Question 8	[redacted]	[redacted]	7	42

Regarding prospective audit and feedback interventions (critical antibiotics prescription review and Longer than 7 days antibiotic prescription review): 49 answers

	Strongly disagree	Somewhat disagree	Somewhat agree	Strongly agree	Not applicable
Question 1	1	1	10	31	6
Question 2	[redacted]	3	12	28	6
Question 3	[redacted]	2	9	32	6
Question 4	[redacted]	3	6	34	6
Question 5b	1	2	8	32	6
Question 6	1	2	6	34	6
Question 7	21	13	5	4	6
Question 8	[redacted]	2	7	34	6
